# Exploring effectiveness of two common trap designs for capturing fish diversity in small freshwater bodies

**DOI:** 10.1007/s10661-026-15133-3

**Published:** 2026-03-16

**Authors:** Kiran Thomas, Milan Gottwald, Daniel Bartoň, Zuzana Šmejkalová, Marek Šmejkal

**Affiliations:** 1 Institute of Hydrobiology, Biology Centre of the Czech Academy of Sciences, České Budějovice, Czech Republic; 2 Faculty of Science, University of South Bohemia, České Budějovice, Czech Republic; 3 Faculty of Agrobiology, Food and Natural Resources, Czech University of Life Sciences Prague, Prague, Czech Republic

**Keywords:** Fish sampling, Biological invasions, Fyke net, Umbrella trap, Freshwater conservation, Conservation monitoring

## Abstract

The biodiversity crisis in freshwater habitats is intensifying due to human-induced changes, leading to significant biodiversity loss. Freshwater fish, in particular, are experiencing alarming population declines. Effective monitoring by direct capture is critical to track and mitigate these losses, but small water bodies often present challenges for active monitoring methods. In such cases, passive sampling techniques, like traps, offer a practical alternative for conservation agencies, though their effectiveness in monitoring fish biodiversity for conservation purposes is not yet fully understood. This study evaluates the effectiveness of two passive sampling techniques fyke nets and umbrella-shaped traps (umbrella traps) for capturing fish diversity in small freshwater habitats. Sampling was conducted overnight in 39 water bodies, and fish density was quantified using catch per unit effort (CPUE). A beta regression model was applied to analyze species capture rate and the influence of habitat parameters on catch success. The CPUE revealed a 6.82-fold higher mean individual capture rate and a 2.05-fold higher species richness per deployment for umbrella traps compared to fyke nets indicating significantly higher sampling efficiency under comparable conditions. Rarefaction curves and non-metric multidimensional scaling analyses demonstrated that umbrella traps more effectively characterized littoral fish diversity and captured higher species richness than fyke nets in small freshwater bodies. The study underscores the utility of umbrella traps as a reliable tool for assessing fish diversity through direct capture, particularly for monitoring elusive or invasive non-native species. These findings aim to enhance conservation oriented species capture and inform conservation planning in often-overlooked small freshwater ecosystems, which play a crucial role in the long-term conservation of fish species.

**Supplementary Information:**

The online version contains supplementary material available at 10.1007/s10661-026-15133-3.

## Introduction

The degradation of freshwater habitats, coupled with unprecedented shifts in fish biodiversity (Moyle & Leidy, 1992; Nagelkerken et al., 2017; Su et al., 2021), underscores the critical need for efficient and reproducible freshwater fish monitoring strategies. Fish are widely regarded as indicator species for evaluating the ecological quality and integrity of freshwater ecosystems, primarily due to their timely responses to both natural and human induced pressures (Blabolil et al., 2017a, b; Fausch et al., 1984; Magurran & McGill, 2010; Naigaga et al., 2011; Schweizer et al., 2022). To develop a proper understanding of fish communities to support conservation efforts in freshwater ecosystems, collecting high-quality data is essential (Blabolil et al., 2017a, 2017b; Kubečka et al., 2009; Říha et al., 2023). This highlights the importance of effective, systematic monitoring of fish populations and communities across diverse freshwater habitats. Special attention must be given to small, often overlooked freshwater bodies, as they face critical pressures from habitat degradation and invasion of non-native species (Oertli & Parris, 2019; Šmejkal et al., 2025). Effective monitoring through direct fish capture can greatly enhance conservation strategies in small freshwater bodies by identifying pure genetic lines for targeted repatriation (Thomas et al., 2025a, b). In general, the objectives of fish species monitoring can vary from confirming the presence of a species, tracking population dynamics of threatened species, to observing changes in community structure over time (Lindenmayer & Likens, 2009; Nichols & Williams, 2006; Radinger et al., 2019; Thomas et al., 2023). Therefore, assessing the effectiveness of monitoring methods and their standardization is essential to enhance the precision of these approaches in achieving their intended objectives.

Given these challenges, locating pure or relict populations for safeguarding genetic diversity of native species (Hvilsom et al., 2022; Minckley et al., 2003; Olden & LeRoy, 2005) and identifying the presence of invasive non-native species are crucial for developing conservation strategies (Fausch, 2006; Olden et al., 2010). These efforts rely heavily on capturing freshwater fish species in their remaining primary habitats and refuge areas (Poos et al., 2007; Radinger et al., 2019). The deterioration and fragmentation of primary habitats, especially those critical for small, specialized freshwater fish, have significantly restricted their natural dispersal capacity, further challenging their presence in such environments (Arthington et al., 2016; Dudgeon, 2014). Additionally, exposure to anthropogenic pressures and invasive non-native species within these habitats further exacerbates their already precarious breeding conditions, hindering the establishment of thriving fish populations (Thomas et al., 2025a, b). Therefore, greater attention should be directed towards identifying areas and extents of fish species occurrences, including small freshwater bodies, and assessing their population viability in situ on a timely basis (Arthington et al., 2016; Brauer & Beheregaray, 2020; Hugueny et al., 2011).

Conservationists and related agencies often work with limited time, personnel, and funding. Although remote and less accessible sites may still harbour pure and relict populations of native fishes, these areas often receive limited attention because of logistical and resource constraints. Small freshwater bodies, and the fish species they support, have long been overlooked and have not been a primary focus of conservation efforts (Biggs et al., 2005). Historically, attention has been directed toward larger aquatic systems such as lakes, rivers, and streams (Hill et al., 2021). As a result, the direct sampling methods developed have primarily been manipulative, tailored to suit the needs of these larger ecosystems. Considering the ecological importance of small and often neglected freshwater habitats, enhancing fish detection and monitoring through direct capture in remote and less accessible sites represents a crucial step toward effective conservation management (Šmejkal et al., 2021, 2025; Thomas et al., 2023, 2025a, b).

Although methods to assess fish diversity have advanced, most still focus on species presence or abundance. Environmental DNA (eDNA) offers a rapid, sensitive, and cost-effective way to detect aquatic species (Harper et al., 2019; Keskin, 2014; Lawson Handley, 2015; Rees et al., 2014). Non-destructive tools like baited and unbaited underwater cameras also enable species monitoring without capture (Ebner & Morgan, 2013; Holubová et al., 2019; Jones et al., 2019; Mallet & Pelletier, 2014). Likewise, underwater visual census (UVC) remains a common approach for in situ assessments of fish richness and abundance (Colton & Swearer, 2010; Edgar et al., 2004; Holubová et al., 2025; MacNeil et al., 2008; Pais & Cabral, 2017). In large freshwater bodies, methods such as seining, gillnetting, trawling, electrofishing, and acoustic surveys are often combined to achieve high detection probability and accurate estimates of species abundance (Blabolil et al., 2017a, b; Jůza et al., 2022; Kubečka et al., 2009; Šmejkal et al., 2015). However, in remote and less accessible habitats where conservation-oriented sampling is necessary, capture methods must be carefully tailored to ensure effectiveness.

When sampling with a conservation-focused approach, a major challenge is that many elusive native fish species are restricted to relatively small and often inaccessible habitats, where anthropogenic pressures and invasive non-native species are typically less prevalent (Castañeda et al., 2020; Leitão et al., 2016). These sites often leave no viable methodological alternatives other than passive techniques for direct capture, particularly those applicable to small floodplain pools. In these small, isolated freshwater habitats, the choice of sampling method is crucial and must be adapted to the habitat characteristics, which can lead to imperfect species detection (Guillera‐Arroita, 2017; Jordan et al., 1997; Merz et al., 2021; Revenga et al., 2005). Sometimes, this challenge is further compounded by the rapid invasion and spread of invasive non-native species. For example, one of the most widespread invasive non-native fish species in Europe is the topmouth gudgeon (*Pseudorasbora parva*) (Gozlan et al., 2010; van der Veer & Nentwig, 2015). Initially introduced unintentionally with aquaculture stocks in the early 1960 s (Bănărescu, 1964), it has since become established throughout much of Europe’s freshwater systems (Gozlan et al., 2010). The gibel carp (*Carassius gibelio*) has increasingly invaded and occupied the primary habitats of the native crucian carp (*Carassius carassius*), gradually displacing them across much of their natural distribution range in Central Europe and the Danube River basin (Šmejkal et al., 2024; Tapkir et al., 2022, 2023). This displacement complicates efforts to locate pure and relict populations of this habitat specialist using conventional passive sampling methods (Thomas et al., 2023). In abandoned quarries with steep slopes and in small ponds or sedimented pools with high macrophyte coverage, conventional direct sampling methods such as electrofishing are ineffective and trapping offers an ideal alternative (Šmejkal et al., 2025; Thomas et al., 2023).

Passive sampling with fyke nets is a traditional method for sampling fish, amphibians, and large water insects in small, less accessible waterbodies, such as flooded stone quarries and ponds with dense macrophytes (Kolar et al., 2021; Sayer et al., 2011; Thomas et al., 2023), and is employed by conservation agencies for fish capture. However, concerns remain about the method’s size selectivity and its overall effectiveness in capturing the full range of species present, leaving its overall reliability in question (Clark et al., 2007; Fische et al., 2010; Turner et al., 2021). Umbrella traps, with their multiple inlet openings positioned in all directions from the trap centre, have proven effective for capturing diverse fish species and amphibians (Thomas et al., 2025a, b; Weber et al., 2023).

In light of these considerations, our objective was to evaluate the effectiveness of two trap designs, the simple fyke net and the umbrella trap in small, often overlooked freshwater habitats, to assess their direct capture efficiency in documenting fish diversity. This aligns with broader goal of detecting remnant pure and relict populations of native crucian carp for potential repatriation and population recovery, as well as enabling the early detection of invasive non-native species within these habitats across the Czech Republic (Šmejkal et al., 2021). We hypothesize that (i) umbrella traps will perform better than fyke nets in capturing the overall fish diversity of the habitat due to its more complex design, (ii) the higher capture rate of species richness in umbrella traps may serve as a strong indicator of this method’s effectiveness in locating elusive species and early detection of invasions in small freshwater bodies, (iii) the inclusion of site parameters may account for variability in detectability beyond the influence of trap type, and (iv) the differences in detection effectiveness between the two trap types could be partially explained by their size selectivity, with umbrella traps potentially targeting larger individuals more effectively than fyke nets.

## Materials and methods

### Design of traps

**Fig. 1:**
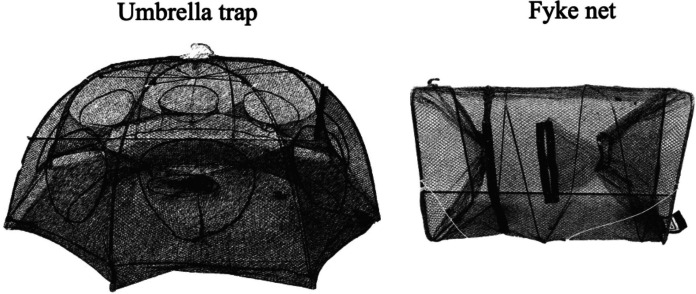
Umbrella shaped trap and fyke net used for sampling the sites

The fyke net size was 25 × 25 × 45 cm, 4 mm mesh size, with two small funnel-shaped openings at opposite ends (6 cm in diameter) and a rope to which a float is attached to pull the net into and out of the water (Fig. 1). The umbrella traps are octagonal in shape (80 × 40 cm; 4 mm mesh size) with eight funnel shaped openings oriented in all directions and a rope to which a float is attached for deployment and retrieval (Fig. 1).

### Sampling

**Fig. 2:**
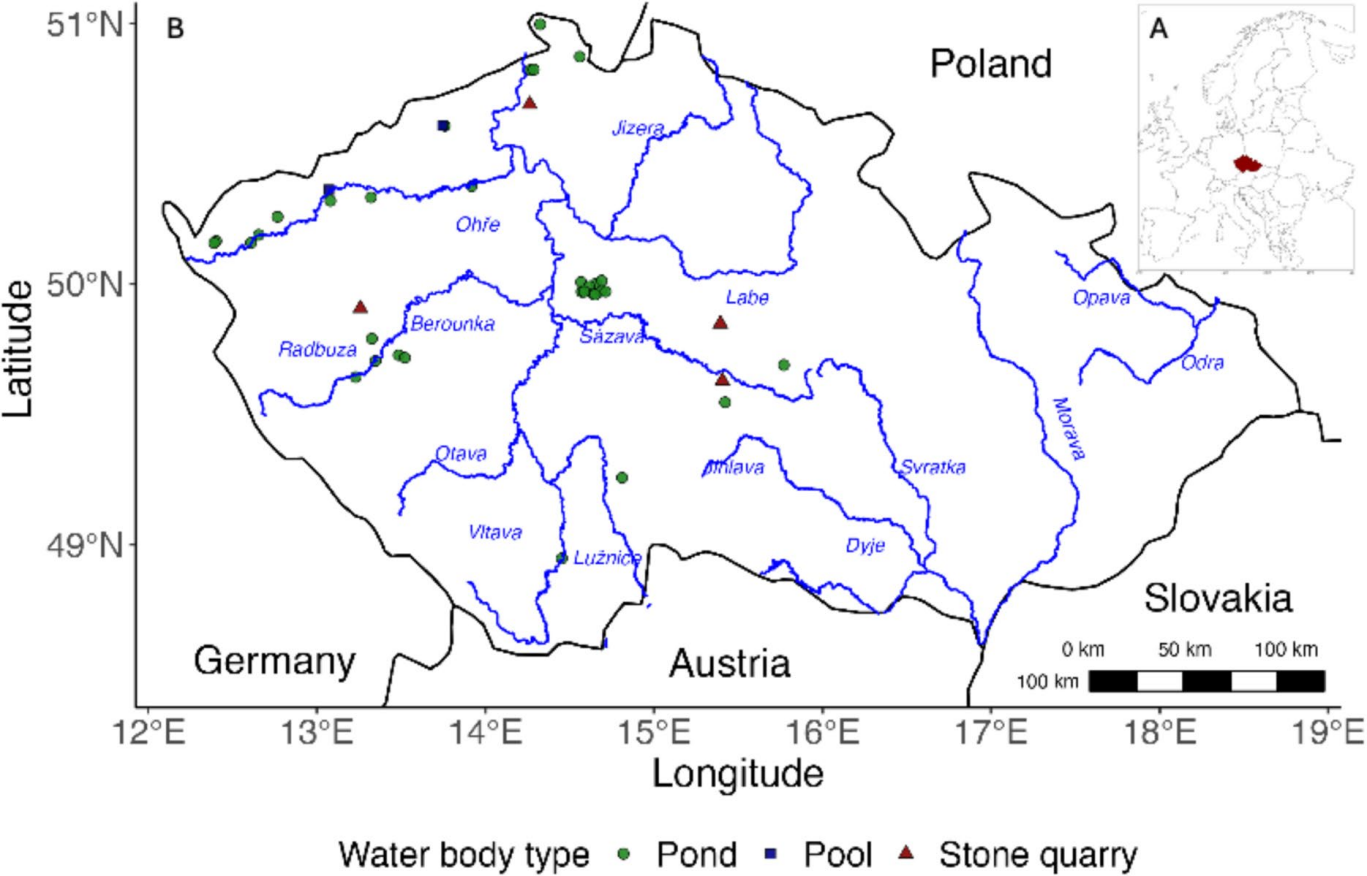
Location map of the sites sampled for fish assemblages using fyke nets and umbrella traps across the Czech Republic. Major river basins are depicted for geographic context. The inset shows the position of the Czech Republic (highlighted in red) within the European Union

The study was conducted at 39 sites across the Czech Republic, all of which were under 2.5 hectares in surface area (an average 0.39 ha of the water area; Table 1) and thus classified as small freshwater habitats (Fig. 2).

**Table 1:** The description of the sites sampled within the Czechia with the geographic location of the site, water body type, macrophyte cover (as percentage of total surface area), area (hectares), transparency (Sneller tube), and elevation of the site in metres are given

Site ID	Water body type	Macrophyte	Area (ha)	Transparency(cm)	Elevation(m)	Environment
1	Pond	0	0.7999	40	391	Forest, field
2	Stone quarry	30	0.0379	41	475	Forest, field
3	Pond	40	0.0434	27.5	520	Forest, field
4	Pond	0	0.09	25	453	Forest, field
5	Pond	0	0.6731	25	438	Forest
6	Pond	0	0.0837	25	295	Urban
7	Pond	0	2.2452	20	170	Field
8	Stone quarry	60	0.0996	30	524	Forest, field
9	Pond	35	0.1239	15	369	Field, urban
10	Pond	0	0.2081	17.5	377	Forest, urban
11	Pond	0	1.9279	25	336	Meadow, urban
12	Pond	25	0.8515	40	419	Forest, urban
13	Stone quarry	17	0.156	15	510	Forest
14	Pond	12	0.9163	25	383	Field, urban
15	Pond	55	0.4104	35	471	Forest, meadow
16	Pond	0	0.2841	28	423	Forest, urban
17	Pond	10	1.3032	25	396	Meadow, urban
18	Pond	10	0.8613	40	465	Forest, meadow
19	Pool	0	0.1112	17	392	Meadow, urban
20	Pond	40	0.1842	25	230	Forest
21	Pool	30	0.0878	14	230	Forest
22	Pond	20	0.5522	16	327	Forest, meadow
23	Pond	50	0.2033	40	321	Forest, wetland
24	Pond	0	0.5034	15	578	Forest, field
25	Stone quarry	10	0.1203	22.5	370	Forest
26	Pond	0	0.1295	25	314	Field, meadow
27	Pond	30	0.4825	20	283	Field, urban
28	Pond	0	0.1734	22.5	349	Urban
29	Pond	2	0.2124	25	370	Field, Urban
30	Pond	0	0.3844	30	355	Urban
31	Pond	30	0.1546	30	349	Urban
32	Pond	10	0.166	65	323	Meadow, urban
33	Pond	15	0.1269	15	348	Urban
34	Pond	34	0.0434	45	310	Wetland, urban
35	Pond	3	0.0414	15	378	Urban
36	Pond	0	0.0284	12.5	373	Urban
37	Pond	4	0.2518	31	410	Forest
38	Pond	10	0.1052	12	422	Urban
39	Pond	83	0.0059	32.5	441	Urban

Sites were sampled during the 2023 growing season, and they fell into three categories: ponds, pools, and former stone quarries. The primary rationale for site selection was the growing challenge of identifying small freshwater habitats that had fish populations and were suitable for trap deployment. Ephemeral pools frequently exhibited water levels that were too low to allow effective trap deployment, and stone quarries, considered secondary habitats for displaced floodplain species such as the crucian carp, were also limited in number. As a result, both habitat types were less represented among the sampling sites. The ponds examined in this study were predominantly disused, man-made water bodies that were not employed for commercial aquaculture. The stone quarries consisted of deep, water-filled basins left behind from previous quarrying activities. The pools were shallow water bodies occurring in river floodplains or surface depressions formed by landslides or clay mining and, were isolated from direct inlets unlike ponds. Site-specific parameters were collected; the percentage of macrophyte cover on the entire water surface area was determined by visual observation. The surface area and elevation of the water body were calculated with the help of online maps (mapy.cz). To measure water transparency, a Sneller tube was used (Solimini et al., 2006; Toivanen et al., 2013).

Combination of dog food pellets and slices of bread were used as bait, based on findings from previous study, to improve the catch success (Jůza et al., 2018; Thomas et al., 2023). A standardized bait quantity of 5 g per trap was used, and the bait was positioned away from the entry points to minimize directional attraction and maintain passive sampling conditions*.* Five traps (three fyke nets and two umbrella traps), followed by Radinger et al. (2019) were deployed overnight in each site. Trap types were deployed within a 10 min window and retrieved within 10 min of each other to ensure consistent exposure duration across traps and eliminate temporal bias. Traps were installed in the littoral zone, approximately 1 m from the shoreline, at depths between 0.5 and 1 m, following a consistent alternating sequence of fyke nets and umbrella traps. To prevent mutual interference, traps were spaced at least 3 m apart and positioned to avoid obstruction by macrophytes, particularly near inlets and outlets. Habitat conditions varied between sites, but intra-site consistency was maintained. Site identity was included in the analysis to account for site-level variability.

Traps were deployed for a total of 3688 h, and the average total deployment time was 18.9 h per waterbody (minimum 14.3 and maximum 26.1). The caught individuals were sorted according to species; the standard length (SL, mm) and weight (W, g) of fish were recorded.

### Data analysis

Catch per unit effort (CPUE) served as a proxy for fish density, calculated as the number of fish of each species caught per hour using trap types. The CPUE was determined separately for each species, each trap, and capture method. As these sites were sampled for the first time, the species present were naive to the trap types. To account for additional variability not related to the trap type, other site-specific parameters were included in the model: waterbody type (pond, stone quarry, or pool), percentage of macrophyte cover, waterbody area, elevation above sea level, and water transparency. These parameters were assumed to potentially influence fish activity and their timidity to enter the novel object, the trap.

#### Species detectability by trap types

The expected number of species for a given sample size was estimated using a hypergeometric distribution model, and rarefaction curves were plotted for trap type catch efforts (Hurlbert, 1971; Sanders, 1968). These curves are used to compare the biodiversity of the sampled communities in terms of species richness, standardized across samples of varying sizes (Gotelli & Colwell, 2001).

Additionally, non-metric multidimensional scaling (NMDS) was used to visualize differences in species composition between samples collected with the two trap types using phyloseq package of R (McMurdie & Holmes, 2013). NMDS was performed on the species abundance matrix using the Bray–Curtis dissimilarity index to account for differences in community structure. The ordination was conducted in a two-dimensional space, minimizing stress to achieve an optimal representation of sample relationships. Barycentres were added for each trap type in the NMDS ordination, and the potential differences between trap types in capturing species diversity across sites were checked by a permutational multivariate analysis of variance (PERMANOVA) (Anderson, 2001), which tests for significant compositional differences between groups. The assumption of homogeneity of multivariate dispersions was verified using the betadisper function of the vegan package (Oksanen, 2015).

#### The effect of site parameters versus trap type on the catch success of the species

An analysis using the beta regression model was implemented as a generalized additive model (GAM) using the mgcv package (R Core Team, 2024; Wood, 2001, 2017) to understand how site parameters affect catch success. Specifically, we examined the effects, trap type (fyke net, umbrella trap), waterbody type (pond, stone quarry and pool), percentage of macrophyte cover, area of the water body, its elevation from the sea level, and water transparency on catch success, measured by CPUE. Interaction effects of trap type and water body type were also included. The model included a site-specific random effect to account for unobserved site-specific influences, capturing variability in catch success across different sites (Nakagawa & Schielzeth, 2013).

#### The effect of the trap types on captured fish size

The potential effect of the trap types on the recorded standard length (SL) of the species captured was analyzed. Normality of SL data was evaluated using the Shapiro–Wilk test, and differences in SL between trap types were subsequently tested using the Mann–Whitney *U* test. The relative sizes of fish species captured were visualized using boxplots and violin plots, presented for each method and sampling site. All computations and graphical outputs were generated using R (R Core Team, 2024).

## Results

**Fig. 3:**
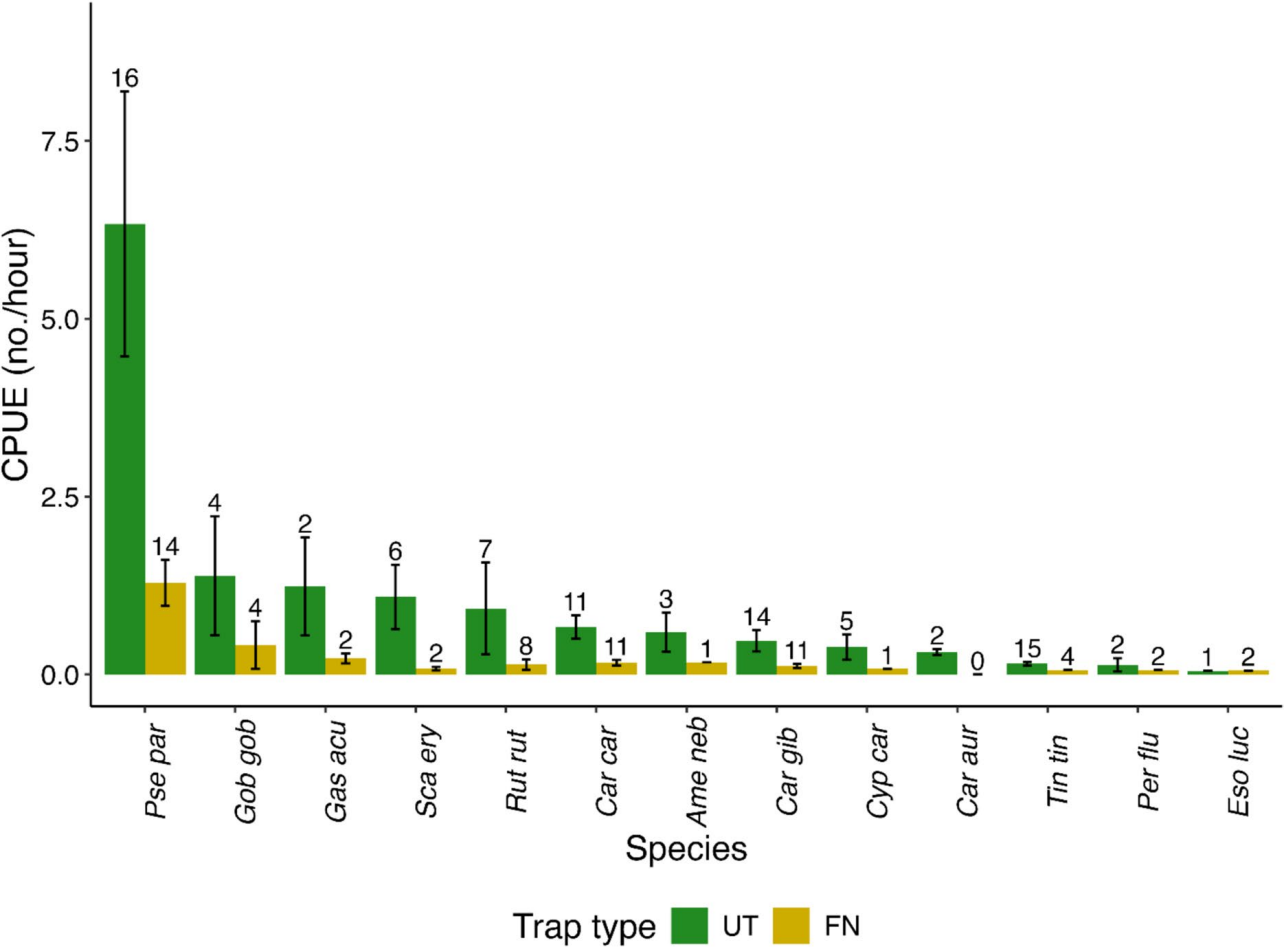
The bar plot depicts the CPUE of those species that are caught by two methods of sampling employed, UT (umbrella traps) and FN (fyke nets). The species are Pse par, topmouth gudgeon (*Pseudorasbora parva*); Gob gob, gudgeon (*Gobio gobio*); Gas acu, stickleback (*Gasterosteus aculeatus*); Sca ery, rudd (*Scardinius erythrophthalmus*); Rut rut, roach (*Rutilus rutilus*); Car car, crucian carp (*Carassius carassius*); Ame neb, brown bullhead (*Ameiurus nebulosus*); Car gib, gibel carp (*Carassius gibelio*); Cyp car, common carp (*Cyprinus carpio*); Car aur, goldfish (*Carassius auratus*); Tin tin, tench (*Tinca tinca*); Per flu, perch (*Perca fluviatilis*); Eso luc, northern pike (*Esox lucius*)

Out of 39 sites involved in the study, thirty-three were ponds, four were former stone quarries, and the remaining two sites were pools (Table 1). The average area of the waterbodies was pond, 0.442 ± 0.530 ha; pool, 0.100 ± 0.017 ha; and stone quarry, 0.103 ± 0.050 ha (Table 1). Total CPUE was generally higher for umbrella trapping, averaging 2.96 individuals (SD = 5.49), and total CPUE for fyke net trapping averaging 0.43 individuals (SD = 0.95) (Wilcoxon test: *W* = 1864.5, *p* < 0.001, Fig. 3). The umbrella traps exhibited a 6.82-fold higher mean individual capture rate and a 2.05-fold higher species richness per trap deployment relative to fyke nets. Although soak durations varied among sites, no statistically significant relationship was observed between soak time and catch success (Pearson correlation analysis, *r* = –0.05, *p* = 0.47, *n* = 194).

### Species detectability by fyke net and umbrella traps

In absolute terms, the fyke net captured a total of 912 individual fish across 12 species, while umbrella trap captured 4232 fish, representing 13 species (Supplementary Table 1). Most species were common across both sampling methods. However, one species goldfish (*Carassius auratus*) was unique to umbrella traps and was not observed in the fyke nets. The species captured and the number of sites where each species was recorded are provided in Supplementary Table 2.

The umbrella trap exclusively captured the invasive non-native gibel carp at six sites, while the fyke net did so at three sites. In the case of the native crucian carp, each trap type uniquely captured it at only one site. A comparison of native crucian carp captures between trap types revealed a statistically significant difference (Wilcoxon signed-rank test, *V* = 59, *p* = 0.023). On average, umbrella traps captured 5.56 fold or times more crucian carp per site than fyke nets. In the case of invasive non-native gibel carp, a statistically significant difference was observed (Wilcoxon signed-rank test, *V* = 103, *p* = 0.016), indicating that umbrella traps captured 4.15-fold more gibel carps per site than fyke nets. A comparison of invasive non-native topmouth gudgeon captures between trap types revealed a statistically significant difference (Wilcoxon signed-rank test, *V* = 126.5, *p* = 0.0027). On average, umbrella traps captured 105 (SD = 146.0) topmouth gudgeons per site compared to 22.8 (SD = 25.5) in fyke nets. Topmouth gudgeon was exclusively captured by umbrella traps at 3 sites, and only by fyke nets at 1 site.

**Fig. 4:**
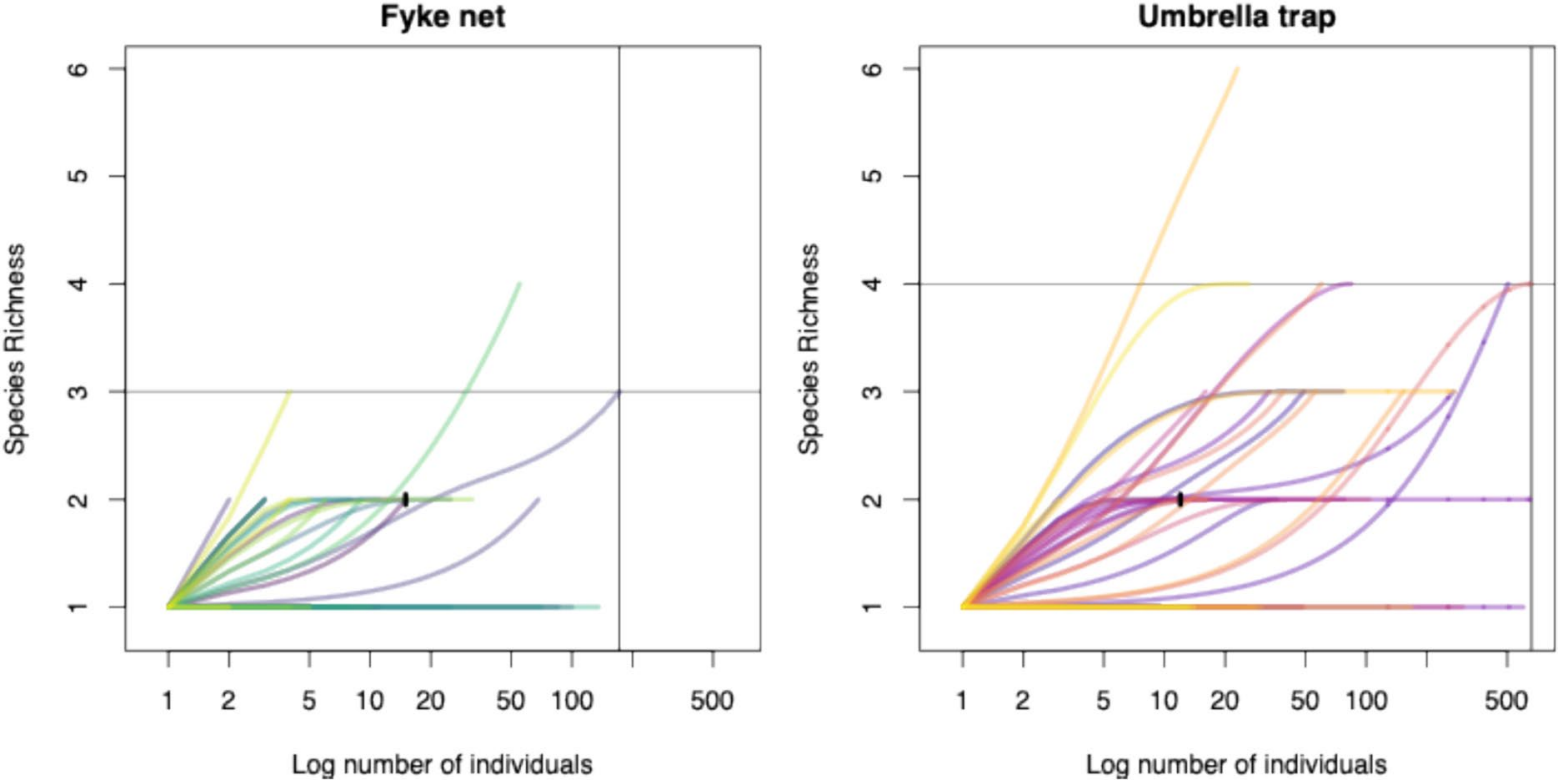
Rarefaction curves showing the number of species caught by fyke netting and by umbrella trapping against the number of individuals caught from each site during the sampling for same duration of sampling effort at a particular site. Each line on the panel represents a site. Steep initial slope indicates that many new species are found with each additional sample. Plateau suggests that most species in the area have been sampled, indicating sampling sufficiency. The umbrella traps with the higher curve at similar sample sizes typically capture greater species richness. The index lines on both the X and Y axis denote benchmark values for total individuals sampled or species saturation thresholds, respectively. These help visualize sampling sufficiency and trap type comparison at equal effort levels

The rarefaction curves demonstrated that umbrella traps caught more individuals among the trap types, which indicates a higher effectiveness with the same sampling effort; the curve extends up to 600 individuals (Fig. 4).

**Fig. 5:**
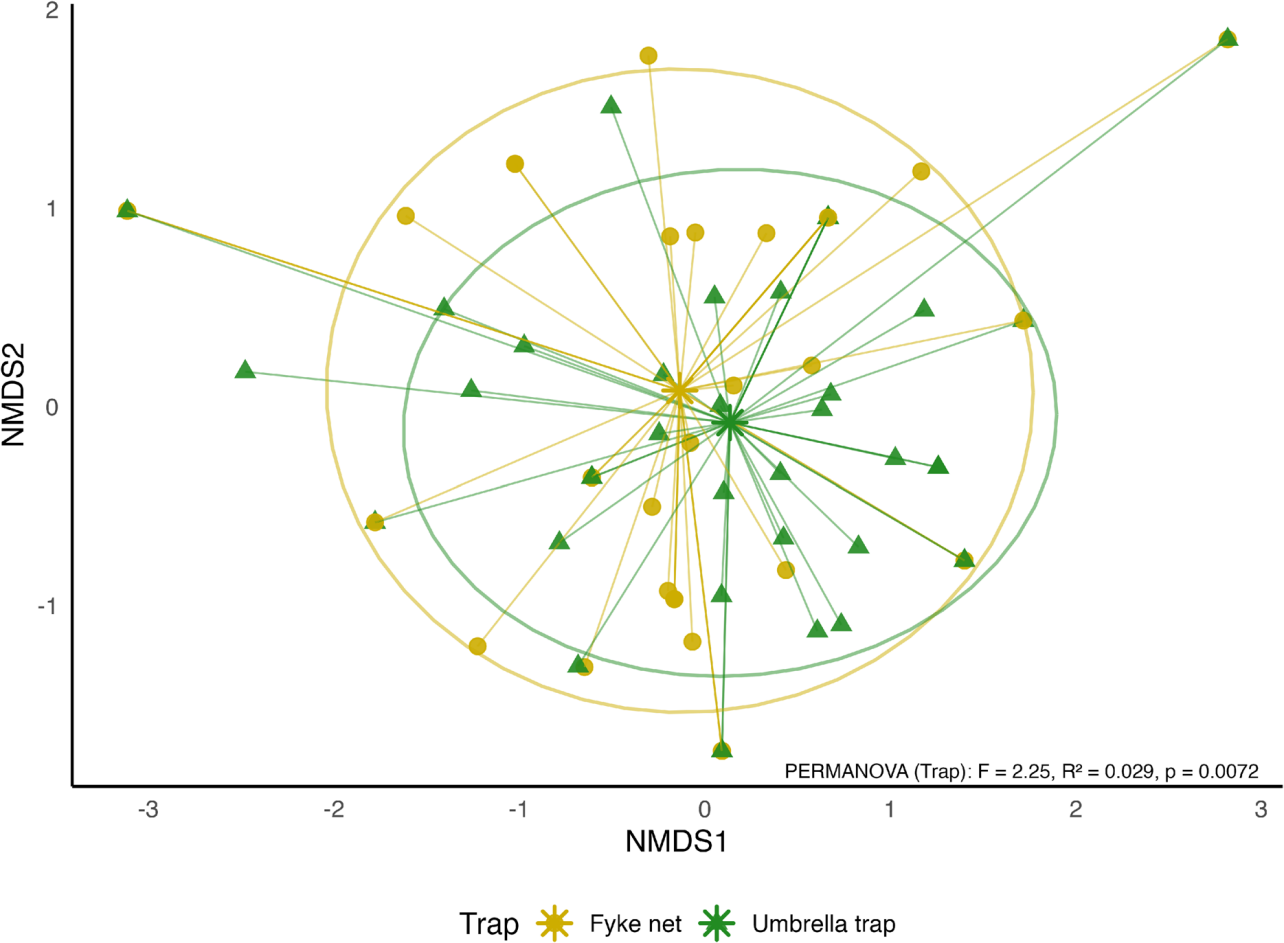
NMDS (Bray–Curtis) ordination of fish assemblages by trap type. Points represent sampling sites, stars denote trap-type barycentres, and shaded ellipses indicate 68% confidence contours. Differences between trap types were tested using PERMANOVA (*F* = 2.25, *R*^2^ = 0.0287, *p* = 0.0072), while multivariate dispersion (betadisper) was non-significant (*p* = 0.872), confirming that the observed separation reflects genuine compositional differences rather than unequal dispersion. The axes represent a rank-based positioning of samples in a reduced dimensional space that best preserves the pairwise dissimilarities between them. Points that are close together represent samples with similar species compositions and points that are farther apart indicate greater differences in species composition

All curves exhibited a steep initial increase, indicating that the first few samples contributed substantially to the observed species richness for both trap types. The curves for the umbrella trap showed a less pronounced plateau at higher sample sizes, suggesting more detailed sampling of the same communities compared to fyke nets (Supplementary Fig. 1). The more gradual increase in species richness corresponding to the number of individuals trapped in the umbrella trap plot indicates that they are more efficient at detecting the presence of multiple species with similar sampling effort compared to fyke nets. The NMDS plot shows an overlap between fyke net and umbrella trap points indicating that both traps capture a similar species composition at the site (Fig. 5).

The NMDS ordination (stress = 0.089) revealed a significant difference in community composition between trap types (PERMANOVA, *F* = 2.25, *R*^2^ = 0.0287, *p* = 0.0072) with similar within-group dispersion (betadisper, *p* = 0.872). The integration of rarefaction analyses and NMDS ordination demonstrates that umbrella traps not only capture a significantly different and broader assemblage of species but also achieve higher species richness at equivalent sampling effort. These findings indicate that umbrella traps provide a more efficient and representative sampling of fish communities in small freshwater habitats compared to fyke nets.

### The effect of site parameters on the catch success

The CPUE model explained 88.7% of the deviance (GAM with a Gamma distribution and log link, *R*^*2*^_*c*_ = 0.754, conditional variance explained by all terms including random effects) indicating a strong overall fit when accounting for both fixed effects and random site-level variation. However, the marginal variance (GAM with a Gamma distribution and log link, *R*^*2*^_*m*_ ≈ 0.00, variance explained by fixed effects only) was effectively zero, suggesting that site-level random effects accounted for the majority of explained variance. Trap type had a significant effect on catch success, with umbrella traps yielding consistently higher CPUE than fyke nets (*p* < 0.001; Table 2). Although an interaction between trap type and water body type was initially included, it was found to be non-significant and subsequently removed.

**Table 2:** Parametric coefficients of catch success in relation to environmental factors: Water body type, trap type, macrophyte cover, transparency, area, elevation, and site-specific random effects (Site.ID), including interaction effects of trap type and water body type

Variable	Estimate	Std. Error	*T* value	*P* value
Intercept	3.73355	1.093081	3.416	0.00143**
Water body type (pool)	0.537902	0.944304	0.57	0.57199
Water body type (stone quarry)	0.393495	0.699634	0.562	0.57684
Trap: UT	2.078499	0.166225	12.504	1.08e − 15***
Macrophyte	0.002311	0.009753	0.237	0.81386
Area (ha)	− 0.270146	0.398416	− 0.678	0.50149
Transparency	− 0.001708	0.017842	− 0.096	0.9242
Elevation (m)	− 0.006807	0.002429	− 2.803	0.00765**
Water body type (pool) × trap type (UT)	− 0.089492	0.695369	− 0.129	0.89822
Water body type (stone quarry) × trap type (UT)	0.331521	0.505554	0.656	0.51558
s(Site.ID)				2e − 16***

The simplified model demonstrated improved or comparable fit (lower generalized cross-validation score and scale estimate) (Table 3), justifying model simplification (*R*^2^_*c*_ = 0.77.5, *R*^2^_*m*_ = −1.03). Among the site-specific covariates, elevation had a statistically significant negative effect on CPUE (*p* < 0.01), with fish abundance decreasing at higher altitudes. Macrophyte cover, area, and water transparency were not significant predictors. A total of 14 sites lacked any macrophyte cover, while only three sites had more than half of the water surface covered by macrophytes. The random effect of site identity was highly significant (*p* < 0.001, Table 3), emphasizing the influence of unmeasured local factors and supporting the inclusion of random effects to account for ecological variability across sites.

**Table 3:** Parametric coefficients of catch success in relation to environmental factors: Water body type, trap type, macrophyte cover, transparency, area, elevation, and site-specific random effects (site ID) simplified model after dropping the interaction effects of trap type and water body type

Variable	Estimate	Std. error	*T* value	*P* value
(Intercept)	3.692975	1.087728	3.395	0.00148**
Water body type: pool	0.499751	0.874145	0.572	0.57047
Water body type: stone quarry	0.570986	0.649535	0.879	0.3842
Trap: UT	2.109754	0.14903	14.157	2e − 16***
Macrophyte	0.002336	0.009711	0.241	0.81099
Area (ha)	− 0.266422	0.396682	− 0.672	0.50538
Transparency	− 0.001302	0.017764	− 0.073	0.94192
Elevation (m)	− 0.00678	0.002418	− 2.804	0.00752**
s(Site.ID)				2e − 16***

### The effect of trap type on the size of captured fish

There was a significant difference in SL between the trap types (Wilcoxon rank sum test with continuity correction, *W* = 66,316, *p* < 0.05). This indicates that the median SL values were not similar between trap types (fyke net, mean = 57.52, SD = 29.64, umbrella trap, mean = 79.19, SD = 45.61). The effect size was estimated at 0.509 (Cohen’s *d*, 95% CI 0.3657 to 0.6526); since the confidence interval does not include zero, the effect is likely real, indicating a significant difference in mean SL between the two trap types. This difference was statistically supported (Supplementary Fig. 2). Species-specific analyses showed that this difference was not uniformly driven by all species. Moderate to large size differences were most evident for species like rudd (Cohen’s *d* = 0.669) and stickleback (Cohen’s *d* = 0.549), which were consistently larger in umbrella traps. In contrast, larger-bodied species such as pike and common carp exhibited small to moderate effects on trap type capture size differences despite their overall body size, likely due to similar SL distributions across trap types and higher variance in umbrella trap captures.

## Discussion

This study highlights the effectiveness of better designed umbrella traps over traditional fyke nets for capturing fish diversity for conservation purposes in small and often overlooked freshwater bodies. It emphasizes the importance of refining sampling strategies tailored to these habitats, favouring umbrella traps over simple fyke nets. The demonstrated effectiveness of umbrella traps underscores their utility in comprehensively capturing species diversity in small freshwater environments (Thomas et al., 2025a, b; Weber et al., 2023). Compared to traditional fyke nets, umbrella traps exhibit superior performance in capturing a broader range of species and much larger sample size for a given species with comparable sampling effort, making them valuable for identifying elusive native species. Furthermore, in contrast to the findings of Thomas et al. (2023), which showed that the invasive non-native gibel carp is often under-represented in fyke nets, umbrella traps have proven more effective in capturing this invasive species as well as topmouth gudgeon. This capability enhances their value for the early detection of invasive species in isolated and less accessible freshwater habitats.

### Species detectability by trap types

Our study observed relatively high catch success across the sites compared to previous observation on the native crucian carp and invasive gibel carp (Thomas et al., 2023). The umbrella trap’s design complexity, characterized by its larger size and eight openings, likely accounts for its superior efficiency, making it a viable method for sampling small and less accessible freshwater bodies. Rarefaction curves demonstrate that when the aim is to estimate the fish diversity of a water body through direct capture, the umbrella trap provides a faster and more effective assessment. However, umbrella traps can catch larger fish compared to fyke nets; it cannot be conclusively stated that they are optimized for detecting larger fish species. This limitation may result in data gaps when assessing the full range of species present. The maximum standard lengths recorded during sampling were approximately 350 mm SL for common carp and 320 mm SL for pike. However, irrespective of species, the greater number of individuals captured by umbrella traps contributed to the observed SL differences between trap types. Thus, if the aim is to discover full range of species present, complementing trapping with other method if feasible or using larger fyke net systems is a possibility (Kubečka et al., 2009). On the other hand, species adapted to floodplain pools, both native and invasive, were captured with good effectiveness (Thomas et al., 2025a, b). The observed decline in fish abundance at higher elevations, as indicated by the GAM, can be largely attributed to the distribution of gibel carp and topmouth gudgeon. These species, which were captured in high numbers from more sites (17 sites each), showed a negative correlation with elevation, a pattern consistent with findings from large-scale study on small stream in Hungary (Takács et al., 2017). However, latitudinal and elevational gradients are well-known to shape fish distribution patterns (Carmona‐Catot et al., 2011; Murphy et al., 2015). Given that macrophytes are widely recognized as key structural components influencing fish abundance and diversity in both lotic and lentic environments (Aarts et al., 2004; de Meo et al., 2023; Holopainen et al., 1997; Neiff et al., 2009; Stauffer et al., 2000), the high density of invasive species across sites may have reduced the observable influence of macrophytes on fish communities and the resulting patterns. The high densities of invasive species such as gibel carp, goldfish, and topmouth gudgeon were found to negatively affect macrophyte coverage (Adámek et al., 2024; Kajgrová et al., 2023; Richardson et al., 1995; Tapkir et al., 2022; Thomaz & Cunha, 2010).

### Effectiveness of the passive traps in small freshwater habitats

The use of passive trapping to assess fish species presence is often debated (Bacheler, 2024; Clavero et al., 2006), highlighting their low efficiency (Jackson & Harvey, 1997; Kubečka et al., 2012), size selectivity (Hubert et al., 2012; Murphy & Willis, 1996), and the potential influence of previously caught fish on capture rates (Bacheler et al., 2013; Conrad et al., 2011; He & Lodge, 1990; Wilson et al., 1993). Despite all this, traps are effective in detecting important changes in the ecosystem over time especially regarding the species assemblage composition transitions (Ilarri et al., 2022; Souza et al., 2024). This study revealed size selectivity between the two trap types, with umbrella traps tending to capture slightly larger individuals. However, umbrella traps also successfully captured smaller individuals, with capture rates nearly as effective as those of fyke nets. Our findings suggest that umbrella traps can serve as a standalone method for direct fish capture in these habitats, as there were no observed instances where fyke nets were relatively more effective (Murphy & Willis, 1996; Ruetz et al., 2007).

When designing a sampling program, understanding the relative selectivity and inherent biases of each sampling method is essential for accurate data interpretation and the development of effective conservation strategies (Zizka et al., 2021). The greater species richness and abundance recorded with umbrella traps compared to fyke nets underscore their reliability as an effective sampling tool, particularly in small or inaccessible freshwater habitats where they represent the only practical method for direct capture.

### Implications for conservation efforts

Overlooked small freshwater habitats should be monitored more effectively to improve our understanding of fish biodiversity and the critical role these habitats play amid the ongoing freshwater biodiversity crisis (Oertli, 2018; Oertli & Parris, 2019; Sayer et al., 2020). Conservation authorities could enhance monitoring strategies through timely upgrades, increasing the effectiveness of detecting native species in vulnerable habitats and enabling early detection of invasive species spread (Chandler et al., 2017; Radinger et al., 2019). In small, less accessible freshwater habitats such as pools and stone quarries, conservation agencies often rely on fyke netting for species detection. However, this method may obscure the presence of certain species (Thomas et al., 2023).

In recent decades, crucian carp populations have suffered significant declines, resulting in their designation as a critically endangered species in the Czech Republic (Chobot & Němec, 2017; Kottelat & Freyhof, 2007). A similar decline is observed in other native freshwater species, such as sunbleak (*Leucaspius delineatus*) (Lelek, 1987; Šmejkal et al., 2025). These population shifts have largely gone unnoticed because small water bodies, critical to their survival, are often overlooked in research and monitoring efforts (Thomas et al., 2025a, b). These trends underscore the urgent need for more effective monitoring of small, often overlooked freshwater habitats. In such cases, umbrella traps can serve as a valuable tool, providing better species information and supporting the development of more effective conservation management strategies.

### Additional factors that may contribute to the reported species pattern and sampling success

Large fishponds and those managed by the Czech Anglers Union, typically used for intensive stocking and recreational fishing, were deliberately excluded from the sampling plan. Preliminary surveys and field observations indicated that such sites were not aligned with the study’s objectives, which focused on stagnophilic fish species (Šmejkal et al., 2021; Thomas et al., 2025a, b). Instead, our sampling focused on small, shallow pools and often abandoned ponds that were nearing the terminal stages of ecological succession, as well as former quarries that lacked a long-standing history of fish stocking or management for recreational angling.

The ponds examined in this study were small, relatively inaccessible, and largely neglected by the public. Given that the sites were sampled for the first time, it is likely that the individuals encountered were naive to the trap, reducing the likelihood of trap avoidance behaviour. These ponds are particularly vulnerable to issues such as cultural eutrophication, habitat degradation, sedimentation, and seasonal drying (Sayer et al., 2010). Furthermore, the study focused on identifying habitats of native crucian carp and invasive non-native gibel carp, with sampling efforts guided by information and reports from local citizens (Thomas et al., 2025a, b). Given these conditions, the full spectrum of fish biodiversity was not expected. For example, the likelihood of detecting rheophilic species was minimal, as these species cannot survive in such stagnant and isolated habitats with frequent anoxia and hypoxia (Daněk et al., 2014; Lujan & Conway, 2015). The limited number of pools and stone quarries restricts the statistical power to draw robust conclusions about habitat-specific differences in CPUE. As such, any observed differences between habitat types should be interpreted with caution. The sampling was carried out across a large geographical area encompassing diverse habitat types. We aimed to deploy the traps overnight at each site to ensure uniform sampling duration. However, in some cases, unexpected circumstances, such as very high fish catches at certain sites, caused delayed retrieval at subsequent sites by a few hours. Despite these challenges, efforts were made to maintain consistency in sampling effort and timing as much as possible across all locations.

The methodological focus was aligned with habitats such as small floodplain pools, an approach consistent with those most actively monitored by the Nature Conservation Agency of the Czech Republic. The sampling plan aimed to target fish species that are morphologically and physiologically adapted to shallow, periodically hypoxic waters with wide temperature fluctuations, conditions characteristic of these pools (Baker et al., 1991; Hoover & Killgore, 2002; Messina & Conner, 1997). Normally, fish species presence has typically been examined with electrofishing, visual estimates, seining, traps, or mark-recapture techniques using the traps or electrofishing (Pess et al., 2005; Roni et al., 2019). At our study sites, standardized use of underwater cameras or visual surveys was not feasible due to the shallow depth and high turbidity of several locations. Electrofishing was also impractical, particularly in steep, inaccessible stone quarries and in ponds or pools nearing the final stages of ecological succession. Although eDNA could theoretically be applied across all sites, our research objectives extended beyond presence–absence data. Specifically, we aimed to collect additional population level information such as body condition and size structure. In this study, baiting was standardized using a combination of dog food pellets and bread. The disparity in the number of each trap type was due to the unavailability of a sufficient number of umbrella traps at the time of the study.

## Recommendations for future work

In future studies, incorporating site variables such as proximity to human populations and the degree of hydrological connectivity is recommended. Additionally, exploring the use of alternative or enhanced attractants could improve catch efficiency and increase the diversity of larger fish species sampled. Rather than analyzing CPUE alone, species richness at each site could be evaluated in relation to environmental variables. Separate analyses for key native and invasive species may further help identify site-specific factors influencing their occurrence. A standardized, nationwide sampling effort that equally represents diverse habitat types would strengthen the conservation of fragmented wetland ecosystems and enhance early detection of invasive species across the Czech Republic.

## Conclusions

In conclusion, our results demonstrate that umbrella traps are a valuable tool for sampling fish species in small, less accessible freshwater habitats for conservation monitoring objectives. Their ability to capture a broader range of species, with comparable sampling effort to conventional fyke nets indicating their potential to provide a more comprehensive understanding of species communities. Umbrella traps, particularly effective for littoral fish species, are well-suited for habitats that support the last remaining populations of native fish species. At the same time, they manage to trap invasive non-native species too. This sampling approach provides a more accurate representation of fish communities and improves the detection of elusive native and invasive species. It thereby supports monitoring and recovery efforts for declining native fish populations, particularly in small freshwater bodies where the use of cutting-edge technologies or combined methods is often impractical.

## Supplementary Information

Below is the link to the electronic supplementary material.

ESM 1 (DOCX 576 KB)

## Data Availability

No datasets were generated or analysed during the current study.
