# Correction to: Exploring effectiveness of two common trap designs for capturing fish diversity in small freshwater bodies

**DOI:** 10.1007/s10661-026-15317-x

**Published:** 2026-04-13

**Authors:** Kiran Thomas, Milan Gottwald, Daniel Bartoň, Zuzana Šmejkalová, Marek Šmejkal

**Affiliations:** 1 Institute of Hydrobiology, Biology Centre of the Czech Academy of Sciences, Česke Budějovice, Czech Republic; 2 Faculty of Science, University of South Bohemia, Česke Budějovice, Czech Republic; 3 Faculty of Agrobiology, Food and Natural Resources, Czech University of Life Sciences Prague, Prague, Czech Republic

**Correction to: Environ Monit Assess (2026) 198:320**

10.1007/s10661-026-15133-3

In the published version of this article, Figure [1, 2 and 4] appeared with insufficient resolution, affecting its clarity. The figure has now been replaced with a higher-resolution version below.


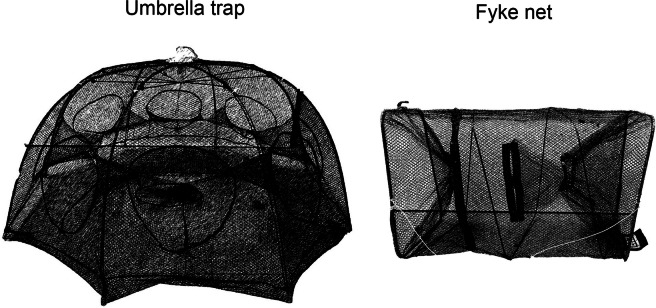
**Fig. 1** Umbrella shaped trap and fyke net used for sampling the sites


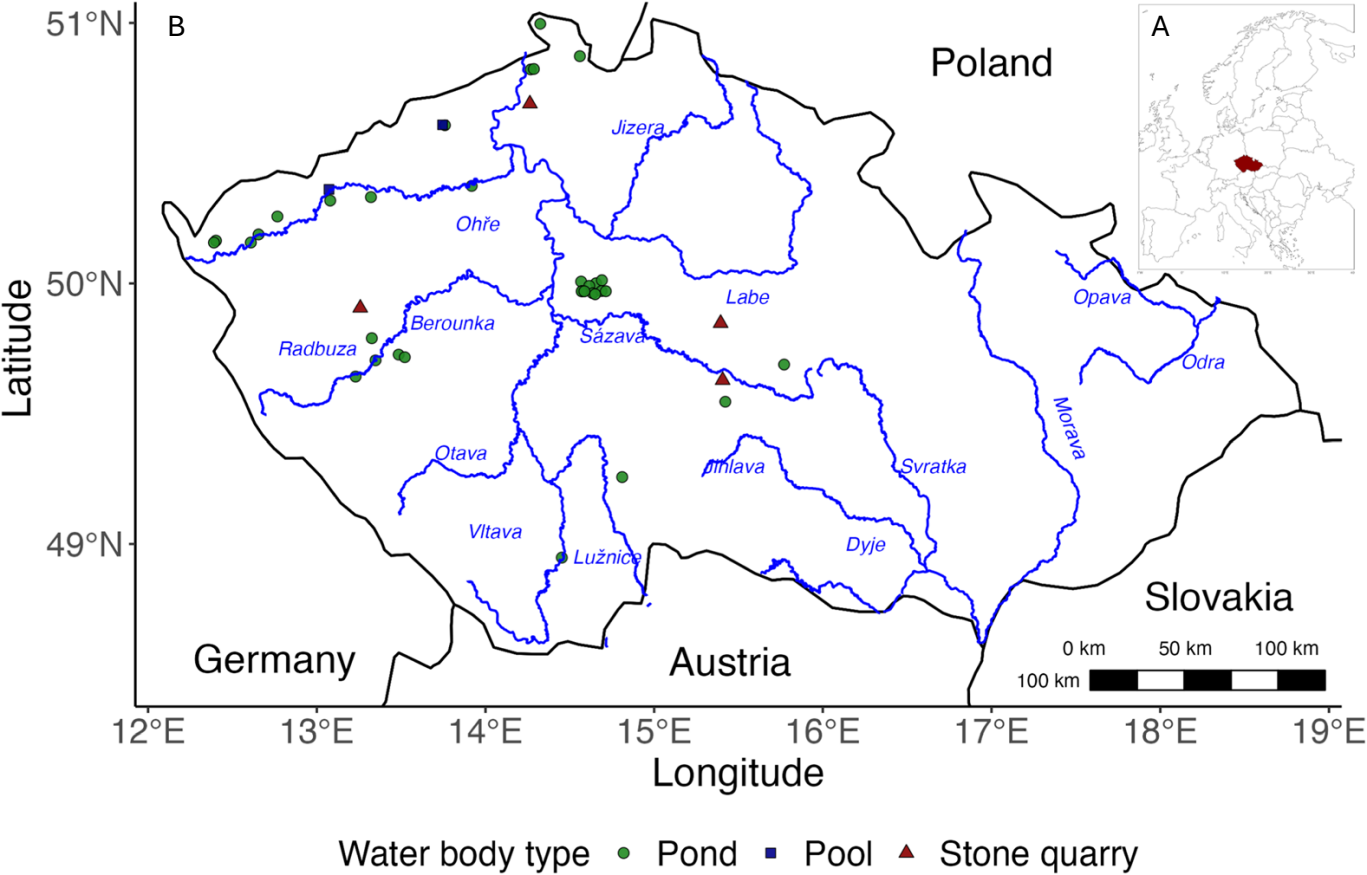
**Fig. 2** Location map of the sites sampled for fish assemblages using fyke nets and umbrella traps across the Czech Republic. Major river basins are depicted for geographic context. The inset shows the position of the Czech Republic (highlighted in red) within the European Union


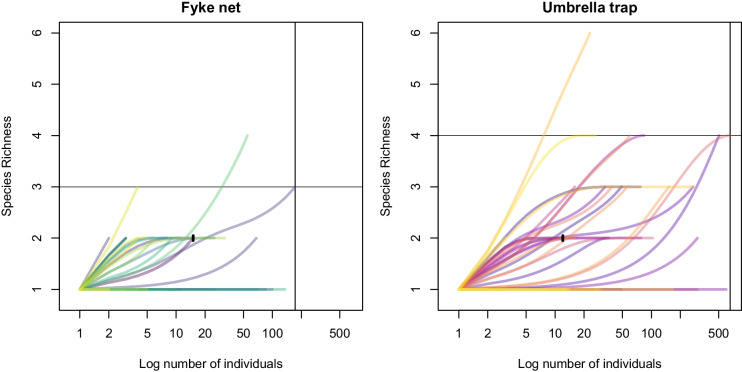
**Fig. 4** Rarefaction curves showing the number of species caught by fyke netting and by umbrella trapping against the number of individuals caught from each site during the sampling for same duration of sampling effort at a particular site. Each line on the panel represents a site. Steep initial slope indicates that many new species are found with each additional sample. Plateau suggests that most species in the area have been sampled, indicating sampling sufficiency. The umbrella traps with the higher curve at similar sample sizes typically capture greater species richness. The index lines on both the X and Y axis denote benchmark values for total individuals sampled or species saturation thresholds, respectively. These help visualize sampling sufficiency and trap type comparison at equal effort levels

