# Supplementary material for: Exploring effectiveness of two common trap designs for capturing fish diversity in small freshwater bodies
Source: Environ Monit Assess. 2026 Mar 16;198(4):320. doi: 10.1007/s10661-026-15133-3 (PMC12992402; doi:10.1007/s10661-026-15133-3)
Supplement: Supplementary file 1 — (DOCX 576 KB) [file 10661_2026_15133_MOESM1_ESM.docx]

Supplementary material to:

Exploring effectiveness of two common trap designs for capturing fish diversity in small freshwater bodies

Kiran Thomas^a,b*^, Milan Gottwald^c^, Daniel Bartoň^a^, Zuzana Šmejkalová^a^ , Marek Šmejkal^a,b^

^a^ Institute of Hydrobiology, Biology Centre of the Czech Academy of Sciences, České Budějovice, Czech Republic

^b^ Faculty of Science, University of South Bohemia, České Budějovice, Czech Republic

^c^ Faculty of Agrobiology, Food and Natural Resources, Czech University of Life Sciences Prague, Prague, Czech Republic

**Supplementary Figure 1:** Rarefaction curves illustrating species richness captured by fyke nets (FN) and umbrella traps (UT) in relation to the number of individuals sampled at each site during the same sampling duration. A steep initial slope indicates that many new species are detected with each additional sample, while a plateau suggests that most species in the area have been recorded, signifying sufficient sampling effort. Only sites with a minimum required number of individuals for the rarefaction curve function are plotted.


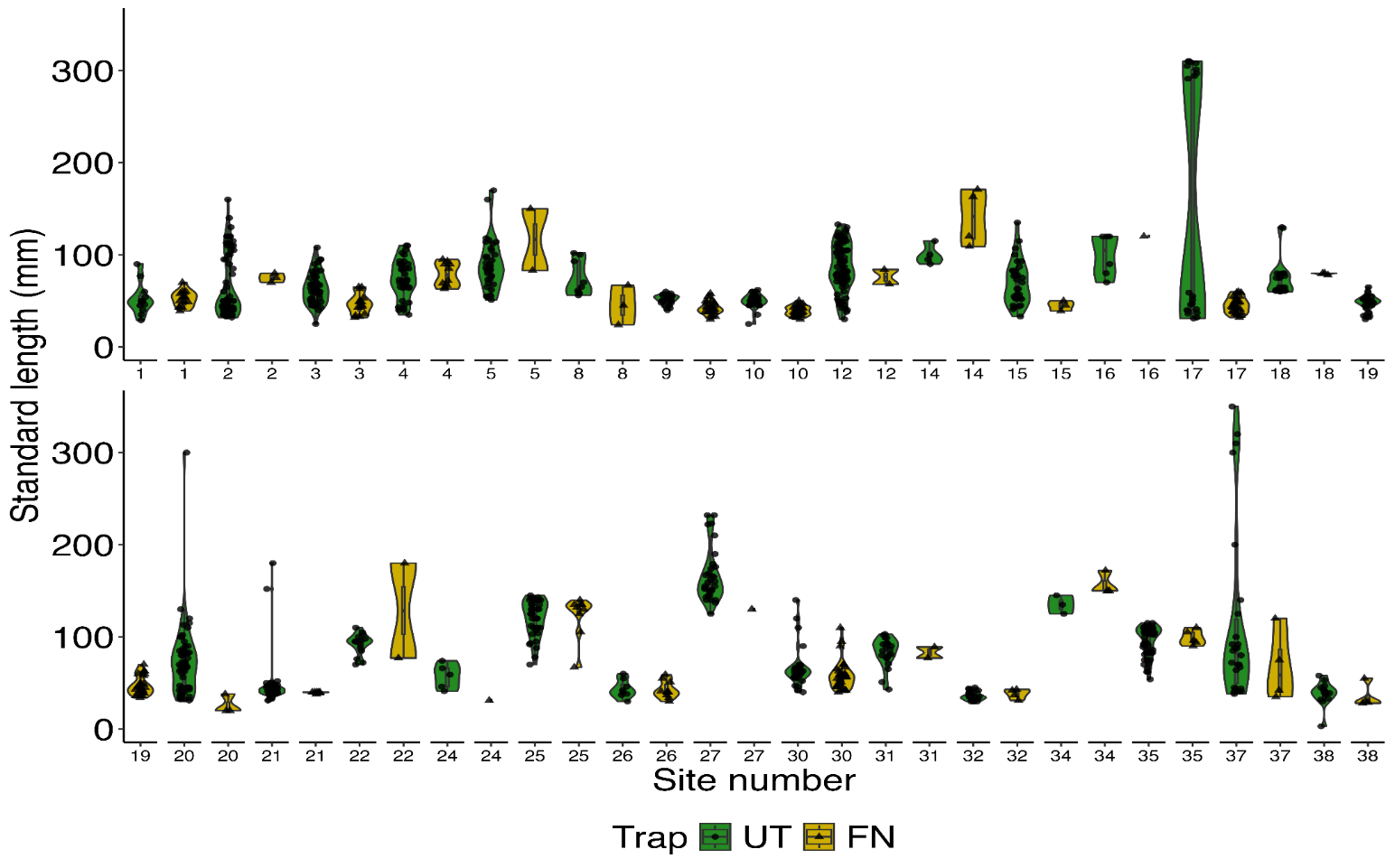


**Supplementary Figure 2:** Violin plots and box plots illustrate the standard-length distribution of fish species caught by fyke nets and umbrella traps. Individual data points used to generate the plots are represented by dots (UT- umbrella traps) and triangles (FN- fyke nets). The analysis includes only those sites where both traps captured sufficient individuals for meaningful comparison. Boxplot is embedded inside to show the median, quartiles and potential outliers. Wider sections of the violin plot indicate a higher probability of individuals having a given standard length, while narrower sections represent lower probabilities.

**Supplementary Table 1:** Summary of the total number of individuals caught and the corresponding number of species detected for both trap types (Fyke Net (FN) and Umbrella Trap (UT)) during the sampling period.

| **Site ID** | **Trap** | **Individuals caught** | **Species caught** |
| --- | --- | --- | --- |
| 1 | FN | 15 | 2 |
| 1 | UT | 12 | 2 |
| 2 | FN | 3 | 2 |
| 2 | UT | 77 | 3 |
| 3 | FN | 15 | 2 |
| 3 | UT | 49 | 3 |
| 4 | FN | 11 | 1 |
| 4 | UT | 48 | 1 |
| 5 | FN | 2 | 2 |
| 5 | UT | 36 | 2 |
| 6 | FN | 68 | 2 |
| 6 | UT | 504 | 4 |
| 7 | FN | 172 | 3 |
| 7 | UT | 271 | 3 |
| 8 | FN | 3 | 2 |
| 8 | UT | 33 | 3 |
| 9 | FN | 100 | 1 |
| 9 | UT | 600 | 1 |
| 10 | FN | 85 | 1 |
| 10 | UT | 301 | 2 |
| 11 | FN | 1 | 1 |
| 11 | UT | 2 | 1 |
| 12 | FN | 2 | 1 |
| 12 | UT | 84 | 4 |
| 13 | FN | 25 | 2 |
| 13 | UT | 78 | 2 |
| 14 | FN | 8 | 2 |
| 14 | UT | 14 | 2 |
| 15 | FN | 3 | 2 |
| 15 | UT | 33 | 2 |
| 16 | FN | 1 | 1 |
| 16 | UT | 6 | 2 |
| 17 | FN | 25 | 1 |
| 17 | UT | 16 | 3 |
| 18 | FN | 2 | 1 |
| 18 | UT | 25 | 1 |
| 19 | FN | 70 | 1 |
| 19 | UT | 300 | 1 |
| 20 | FN | 3 | 1 |
| 20 | UT | 103 | 2 |
| 21 | FN | 8 | 1 |
| 21 | UT | 49 | 1 |
| 22 | FN | 11 | 2 |
| 22 | UT | 17 | 2 |
| 23 | FN | 135 | 1 |
| 23 | UT | 659 | 4 |
| 24 | FN | 1 | 1 |
| 24 | UT | 5 | 1 |
| 25 | FN | 9 | 2 |
| 25 | UT | 39 | 3 |
| 26 | FN | 10 | 1 |
| 26 | UT | 8 | 1 |
| 27 | FN | 1 | 1 |
| 27 | UT | 60 | 4 |
| 28 | FN | 3 | 2 |
| 28 | UT | 56 | 3 |
| 29 | FN | 1 | 1 |
| 29 | UT | 16 | 2 |
| 30 | FN | 55 | 4 |
| 30 | UT | 153 | 3 |
| 31 | FN | 2 | 1 |
| 31 | UT | 30 | 1 |
| 32 | FN | 4 | 1 |
| 32 | UT | 28 | 1 |
| 33 | FN | 6 | 2 |
| 33 | UT | 23 | 6 |
| 34 | FN | 32 | 2 |
| 34 | UT | 274 | 3 |
| 35 | FN | 5 | 1 |
| 35 | UT | 167 | 1 |
| 36 | FN | 5 | 2 |
| 36 | UT | 3 | 2 |
| 37 | FN | 4 | 3 |
| 37 | UT | 26 | 4 |
| 38 | FN | 4 | 2 |
| 38 | UT | 14 | 1 |
| 39 | FN | 2 | 1 |
| 39 | UT | 13 | 1 |

| Common name | Scientific name | Number of sites |
| --- | --- | --- |
| Brown bullhead | *Ameiurus nebulosus* | 3 |
| Crucian carp | *Carassius carassius* | 12 |
| Common carp | *Cyprinus carpio* | 5 |
| Gibel carp | *Carassius gibelio* | 17 |
| Gudgeon | *Gobio gobio* | 4 |
| Goldfish | *Carassius auratus* | 2 |
| Perch | *Perca fluviatilis* | 4 |
| Northern pike | *Esox lucius* | 2 |
| Roach | *Rutilus rutilus* | 10 |
| Rudd | *Scardinius erythrophthalmus* | 7 |
| Stickleback | *Gasterosteus aculeatus* | 2 |
| Tench | *Tinca tinca* | 15 |
| Topmouth gudgeon | *Pseudorasbora parva* | 17 |

**Supplementary Table 2:** Summary of the species captured (alphabetical order) and number of sites the species was captured during the sampling period.
